# Mandibular Anterior Nutrient Canals in Periapical Radiography in Relation to Hypertension

**DOI:** 10.5812/numonthly.15292

**Published:** 2013-11-04

**Authors:** Morteza Abdar-Esfahani, Mojdeh Mehdizade

**Affiliations:** 1 Department of Cardiology, Faculty of Medicine, Isfahan University of Medical Sciences, Isfahan, IR Iran; 2 Department of Radiology, Faculty of Medicine, Isfahan University of Medical Sciences, Isfahan, IR Iran

**Keywords:** Haversian System, Hypertension, Radiography, Mandible

## Abstract

**Background:**

Hypertension (HTN) is the most commonly encountered systemic disease in general population. Nutrient canals contain blood vessels and nerves that mostly appear in the anterior mandibular region. Nutrient canals are not detected in radiographs of all patients, and their normalcy is controversial by many investigators.

**Objectives:**

The present study investigated the correlation between the appearance of nutrient canals and hypertension as a potential clue to diagnose patients with hypertension.

**Patients and Methods:**

Thirty two patients with HTN and 32 normotensive subjects were selected. Periapical radiographs were taken from mandibular anterior cuspid-central incisor region since nutrient canals are commonly observed in this area. Data was analyzed by SPSS software with Chi-square and Fisher tests.

**Results:**

The incidence of nutrient canals was 37.5% in patients with hypertension and 53.1% in the normotensive subjects, but this difference was not statistically significant (P = 0.209). We did not find any association between duration of hypertension (P = 0.292) or controlled hypertension (P = 0.144), and the presence of nutrient canals. The mean of subject age with nutrient canal was more than those without nutrient canals, and this difference was statistically significant in normotensive patients.

**Conclusions:**

This study revealed that there was no significant association between mandibular anterior nutrient canals and hypertension.

## 1. Background

Nutrient canals contain blood vessels and nerves that mostly appear in mandibular anterior region. Nutrient canals have a vertical direction rather than horizontal, and also have been called vascular channels, circulatory canals or interdental canals (1). Some investigators believed the presence of nutrient canals in mandibular anterior region as normal anatomic features, but some others reported radiographic appearance of nutrient canals as pathologic conditions. According to the findings, some pathologic conditions presumably correlated with radiographic appearance of nutrient canals were periodontal disease, hypertension, diabetes, tuberculosis, rickets, calcium deficiency, disuse atrophy, and coarctation of aorta (2). Hypertension is an important health problem which increases the risk of developing cardiovascular disease, stroke and renal disease (3, 4). Hypertension is the most commonly encountered systemic disease in general population. Dental services in patients with hypertension require special care (5). Hypertension is virtually without symptoms, and is usually detected randomly when patient visits his or her physician due to other reasons. Thus, it could be important to discover some clues for the diagnosis of hypertension.

## 2. Objectives

Nutrient canals are not detected in radiographs of all patients and their normalcy is controversial by many investigators (6). Recent studies suggested that further investigations are needed to clear the association between the presence of nutrient canals and systemic disease such as hypertension (7). The present study investigated the correlation between the appearance of nutrient canals and hypertension.

## 3. Patients and Methods

### 3.1. Participants

This is a case-control study conducted on patients with hypertension and subjects without evidence of blood pressure disease. Patients with hypertension were chosen from those who referred to cardiovascular research center in Isfahan city, Iran. Normotensive subjects were chosen from those who referred to dental school of Isfahan University of Medical Sciences, Iran for dental problems. Patients with diabetes, periodontal disease, tuberculosis, rickets and pregnant women were excluded. Sixty four participants were selected aged between 30 to 55 years. Thirty two patients with HTN confirmed by a cardiologist, and 32 normotensive subjects were chosen by simple randomization.

Sample size was calculated by the following equation (8):

$$n={\left(z_{1-\frac{\alpha }{2}}+z_{1-\beta }\right)}^{2}\mathrm{[P}1(1-P_{1})+P_{2}(1-P_{2})]/d^{2}$$

d = 0.3 , P_2_ = 0.35 , P_1_ = 0.05 , β = 0.1 , α = 0.05

The relevant ethics committee of Isfahan University of Medical Sciences, Isfahan, Iran approved the research (project number: 82100). Informed consent form was completed by the subjects.

### 3.2. Radiography

Periapical radiographs were taken by using the Kodak E-speed films and the long cone trophy bisecting angle technique and x-ray unit (70 kilo voltage peak and 10 milliamperes) from the mandibular anterior cuspid-central incisor region since nutrient canals are commonly observed in this area. The radiologists were blinded to the clinical status of normotensive and hypertensive subjects. Radiographic findings for each participant were recorded on special forms.

### 3.3. Assessment of Blood Pressure

Systolic and diastolic blood pressures were measured by a standardized mercury sphygmomanometer on the right arm. Subjects were asked to sit in a comfortable place for 15 minutes. Before measuring the blood pressure, drinking tea or coffee, smoking, full bladder and physical activity were assessed. The systolic blood pressure was defined as the appearance of the first sound (Korotkoff phase 1), and diastolic blood pressure was defined as the disappearance of the sound (Korotkoff phase 5). Blood pressures higher than 140/90 mmHg was defined as hypertension, and less than 140/90 mmHg as normal (9).

### 3.4. Statistical Analysis

SPSS software (version 21) was used for statistical analysis. Chi square test and fisher test were applied for assessing the presence of nutrient canals in normotensive and hypertensive subjects. P < 0.05 was considered statistically significant.

## 4. Results

This study was conducted on 32 patients with HTN and 32 normotensive subjects. There was 34 females (53.12%) and 30 males (46.87%). Basic characteristics of participants in the two groups are shown in Table 1. The incidence of nutrient canals was37.5% in patients with HTN, and 53.1% in the normotensive subjects, but this difference was not statistically significant (P = 0.209) (Table 2). 

**Table 1.** Characteristics of Participants

	Hypertensive Patients	Normotensive Subjects	P Value
**Body mass index, mean ± SD, kg/m^2^**	23.2 ± 2.77	22.5 ± 2.95	0.71
**FBS^a^, mean ± SD, mg/dL**	103.0 ± 12.1	101.8 ± 17.0	0.9
**TC^a^, mean ± SD, mg/dL**	194.2 ± 12.87	184.4 ± 37.46	0.59
**TG^a^, mean ± SD, mg/dL**	149.6 ± 5.94	147.2 ± 5.06	0.51
**HDL^a^, mean ± SD, mg/dL**	42.0 ± 2.38	43.7 ± 2.27	0.41
**LDL^a^, mean ± SD, mg/dL**	114.4 ± 1.37	107.0 ± 5.09	0.09

^a^ Abbreviations: FBS, fasting blood sugar; TC, total cholesterol; TG, triglyceride; HDL, high density lipoproteins; LDL, low density lipoproteins.

**Table 2.** Incidence of Nutrient Canals in Normotensive and Hypertensive Patients

Nutrient Canal	Normotensive, No. (%)	Hypertensive, No. (%)	Total, No. (%)	P Value
**Present**	17 (53.1)	12 (37.5)	29 (45.3)	0.209
**Absent**	15 (46.9)	20 (62.5)	35 (54.7)	
**Total**	32 (100)	32 (100)	64 (100)	

We separately analyzed incidence of nutrient canals in hypertensive male and female (Table 3). Findings showed that there wasno significant association between the incidence of nutrient canals and genders. In addition, there was no significant association between the incidence of nutrient canals in normotensive male and female (P = 0.804).

**Table 3.** Incidence of Nutrient Canals in Hypertensive Male and Female

Nutrient Canal	Hypertensive Male	Hypertensive Women	Total	P Value
**Present**	9 (39.1)	3 (33.3)	12 (37.5)	0.761
**Absent**	14 (60.9)	6 (66.7)	20 (62.5)	
**Total**	23 (100)	9 (100)	32 (100)	

Patients with HTN and normotensive subjects who had nutrient canal were older than those without nutrient canals. In normotensive subjects, the mean age of subject with nutrient canal was more than those without nutrient canals statistically significantly (Table 4). In whole population, the mean age in participants with nutrient canals (47.1 ± 3.2) was 5 years more than those without nutrient canals significantly (42.6 ± 5.1) (P = 0.002).

**Table 4.** Mean of Age in Normotensive and Hypertensive Patients

	Nutrient Canal		P Value
	Present	Absent	
**Patients with HTN^a^**	48.8 (3.8)	45.1 (4.2)	0.06
**Normotensive subjects**	45.9 (6.9)	39.2 (8.3)	< 0.001

^a^ Abbreviation: HTN, hypertention.

We did not find any association between duration of hypertension (P = 0.292) or controlled hypertension (P = 0.144) and the presence of nutrient canals.

## 5. Discussion

We found that frequency of nutrient canals in anterior segment of mandible in patients with HTN was more than those normotensive participants. However, there was no significant difference between the two groups. The mean age of subject with nutrient canal was more than those without nutrient canals, and this difference was statistically significant in normotensive patients. There was no significant association between sex and nutrient canals.

Yilmaz et al. (10) reported that nutrient canals were not indicative for hypertension. In addition, Patni et al. did not show any association between nutrient canals and hypertension (11). Others have also indicated that the presence of nutrient canals did not correlate significantly with systemic diseases such as hypertension (7). However, some studies showed a positive correlation between nutrient canals and systematic disease (11-13). Mani et al. (2) indicated that the prevalence of nutrient canals in patients with HTN was significantly more than control group. They reported that nutrient canals could be used as a clue to diagnose patients with HTN. Other studies confirmed these findings (11, 13, 14).

In the current study, the association of nutrient canals and hypertension was not sex-dependent. However, Patsakas et al. (14) reported that nutrient canals were more frequent in hypertensive males than females. Mani et al. (2) found a higher prevalence of nutrient canals in hypertensive females than males.

Our results showed that aging leads to an increase in the presence of nutrient canals. Some studies similar to our findings found a positive association between prevalence of nutrient canals and increasing age (11, 15, 16). Bilge et al. (12) indicated that prevalence of nutrient canals in older participants was more than younger ones.

In conclusion, this study revealed that there was no significant association between mandibular anterior nutrient canals and hypertension. However, increasing age might increase the presence of nutrient canals.
